# Journey on Naphthoquinone and Anthraquinone Derivatives: New Insights in Alzheimer’s Disease

**DOI:** 10.3390/ph14010033

**Published:** 2021-01-05

**Authors:** Marta Campora, Valeria Francesconi, Silvia Schenone, Bruno Tasso, Michele Tonelli

**Affiliations:** Dipartimento di Farmacia, Università degli Studi di Genova, Viale Benedetto XV, 3, 16132 Genova, Italy; campora@difar.unige.it (M.C.); francesconi.phd@difar.unige.it (V.F.); schenone@difar.unige.it (S.S.); tasso@difar.unige.it (B.T.)

**Keywords:** naphthoquinone/anthraquinone derivatives, Alzheimer’s disease (AD), Aβ aggregation inhibition, AChE and BChE inhibition, Tau inhibition, multitarget directed ligands

## Abstract

Alzheimer’s disease (AD) is a progressive neurodegenerative disease that is characterized by memory loss, cognitive impairment, and functional decline leading to dementia and death. AD imposes neuronal death by the intricate interplay of different neurochemical factors, which continue to inspire the medicinal chemist as molecular targets for the development of new agents for the treatment of AD with diverse mechanisms of action, but also depict a more complex AD scenario. Within the wide variety of reported molecules, this review summarizes and offers a global overview of recent advancements on naphthoquinone (NQ) and anthraquinone (AQ) derivatives whose more relevant chemical features and structure-activity relationship studies will be discussed with a view to providing the perspective for the design of viable drugs for the treatment of AD. In particular, cholinesterases (ChEs), β-amyloid (Aβ) and tau proteins have been identified as key targets of these classes of compounds, where the NQ or AQ scaffold may contribute to the biological effect against AD as main unit or significant substructure. The multitarget directed ligand (MTDL) strategy will be described, as a chance for these molecules to exhibit significant potential on the road to therapeutics for AD.

## 1. Introduction

Alzheimer’s disease is deemed by World Health Organization as one of the most common neurodegenerative diseases and more than 80% of total dementia cases in elderly people. In 2019 World Alzheimer Report estimated over 50 million people living with dementia globally, a figure set to increase to 152 million by 2050 [1]. The clinical manifestations of AD are characterized by misfunctioning and gradual neuronal death, resulting in a progressive memory deterioration and cognitive decline, related to the loss of cholinergic and glutamatergic function. The two distinctive hallmarks of AD are the presence of extracellular accumulated Aβ plaques [2] and hyperphosphorylated tau protein in the form of intracellular neurofibrillary tangles (NFT) [3]. AD pathogenesis is not yet fully understood, even if during the years different hypotheses have been formulated; currently it is usually described as a multifactorial disease caused by several factors which include: loss of cholinergic transmission, excessive protein misfolding and Aβ aggregation [4,5], oxidative stress and free radical formation [6], metal dyshomeostasis [7], excitotoxicity, and neuroinflammatory processes [6]. Moreover, the range of targets in AD is increasing, and for most part, enzymes have been recognized as crucial partners to AD onset and progression [8]. Hence, a number of molecules has entered clinical phase study with their targets, such as β-secretase (BACE1), phosphodiesterase, phospholipase A2, mitogen-activated protein kinase (MAPK) and sirtuin 1 (SIRT1), as an example (clinicaltrials.gov). 

Bulk of evidence sheds light on the interconnected role played by these factors in AD pathogenesis and, consequently, on the difficulties of setting up more effective drugs over current therapies. The marketed drugs for the treatment of AD, namely the acetylcholinesterase (AChE) inhibitors donepezil, rivastigmine, and galantamine and the NMDA receptor antagonist memantine, are regarded as merely symptomatic, respectively modulating the cholinergic or glutamatergic function [9]. However, the synergistic effect between donepezil and memantine in combination regimen is showing ameliorated outcomes for cognition, global assessment, daily activities, and neuropsychiatric symptoms, but lower acceptability than monotherapy [10].

**Figure 1:**
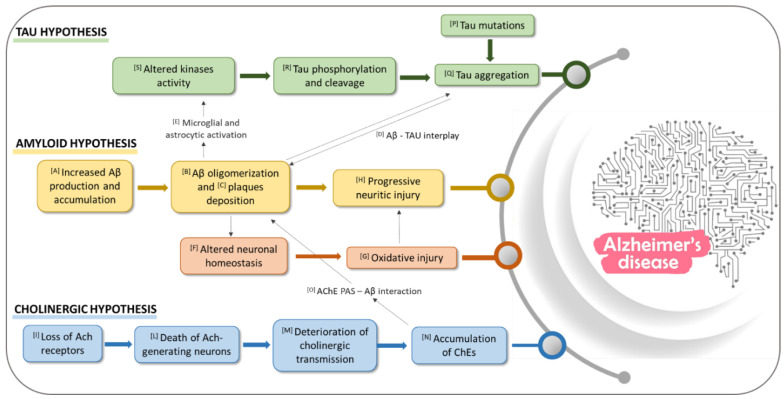
Schematic representation of some biochemical pathways implicated in AD pathogenesis: the interconnection between main targets of NQ and AQ derivatives. Each letter refers to a key event that signs AD etiology, as detailed in the following paragraphs (Section 1.1, Section 1.2 and Section 1.3).

In this scenario, the medicinal chemistry efforts have been paid with a view to disclosing novel chemotypes which could include in their structures the required pharmacophoric features to target one or more of these factors implicated in AD. Many examples of NQ and AQ compounds from natural sources or synthetic have emerged in virtue of their promising properties against AD. In the present review we will focus our survey on cholinesterases, Aβ and tau proteins as main targets of NQ and AQ compounds, whose networked roles in AD etiology are detailed as follows (Figure 1).

### 1.1. Role of Aβ in AD

β-amyloid is a protein consisting in 40–42 amino acids, formed by proteolytic cleavage of a 695 amino acids long type I transmembrane protein, known as amyloid precursor protein (APP) [11]. This proteolytic cleavage can take two different pathways. In physiological conditions, it occurs via the major non amyloidogenic pathway involving an α-secretase that cleaves APP to form soluble α-APP, which is removed from the brain, and a membrane-tethered intracellular C-terminal fragment, called CTFα or C83 [12]. A second enzyme, γ-secretase, located within the transmembrane zone, then cleaves the membrane peptide into two small peptides, p3 and APP intracellular domains (AICDs), which are not “amyloidogenic” [12,13] The process of APP cleavage has been shown to be impaired in genetically determined forms of AD [14,15]. The amyloidogenic pathway begins with the cleavage of the extracellular part of APP by β-secretase, which forms soluble APPβ fragment (sAPPβ) and a C-terminal fragment CTFβ or C99 [12]. This process is followed by the formation of pathological β-amyloid (Aβ_40_ and Aβ_42_) by γ-secretase [13], which accumulates in the brain forming cellular fibrillar deposits known as amyloid plaques [16]. The amino acids sequence of Aβ peptide was discovered in 1984, from extracellular deposits and amyloid plaques [17]. The Aβ_40_ peptide contains 17 hydrophobic, 11 polar and 12 charged residues [18]. Aβ_42_ peptide includes two additional hydrophobic residues at the C-terminal residue, which makes the Aβ_42_ peptide more toxic and aggregation prone [19]. Biophysical studies suggest that the Aβ peptide sustains a series of transitions, from a structure rich in α–helix to one in which β-strands prevail. The two forms of Aβ have distinct biological activity and behavior at the earliest stage of assembly. Studies of the kinetics of Aβ fibril formation have shown that Aβ_42_ forms fibrils much faster than Aβ_40_ [20,21]. Aβ_42_ is more fibrillogenic and more neurotoxic than Aβ_40_. The initial phase of oligomerization of Aβ_42_ monomers involves the formation of pentamer/hexamer units, so-called paranuclei [22]. Paranuclei are initial and minimal structures that can oligomerize to larger forms, namely large oligomers, protofibrils, fibrils. Monomers, paranuclei and large oligomers are predominately unstructured with only short β-sheet/β-turn and helical elements. During protofibril formation essential conformational changes occur when the unstructured, α-helix, and β-strand elements transform into β-sheet/β-turn structures. Paranuclei could not be observed for Aβ_40_ at similar concentrations of the peptide [23]. Until recently, the fibrillar Aβ_40_ and Aβ_42_ were considered the only toxic forms of this peptide, but it is now clear that Aβ oligomers and protofibrils are more neurotoxic than mature Aβ fibrils or amyloid plaques. Targeting the Aβ peptide cascade has been at the heart of therapeutic development in AD research since its formulation in 1992 [24], even if drugs based on this hypothesis have not reached commercialization yet. Essentially, there are three different ways to approach Aβ as a therapeutic strategy. The first is based on the limitation of Aβ production (Figure 1[A]) through the inhibition of β- and γ-secretase or the activation of α-secretase [25,26,27]. The second lies in inhibiting Aβ oligomerization and fibrillization and/or destabilizing preformed Aβ fibrils (Figure 1[B]) [25]. The last focuses on the regulation of Aβ levels through targeting Aβ clearance (Figure 1[C]), which is mediated by two distinct mechanisms, its hydrolysis by cerebral proteases, both intra and extracellular, and independently by transport from the brain and subsequent proteolytic removal in the periphery [28]. Evidences have demonstrated the role of tau as crucial partner of Aβ in AD pathogenesis [29]. Moreover, the intracellular binding of soluble Aβ to non-phosphorylated tau was detected, and possibly described as a precursor event to later self-aggregation of both molecules (Figure 1[D]) [30]. Aβ, activated microglia and astrocyte have been also shown to affect tau pathology through the upregulation of kinases and pro-inflammatory cytokines that modulate tau phosphorylation (Figure 1[E,S]) [31,32]. 

The neurotoxicity of the prefibrillar aggregates appears to result from their ability to trigger a whole cascade of harmful mechanisms, including the neuroinflammatory process, oxidative stress, and excitotoxicity, which leads ultimately to loss of synapses, intraneuronal connections, and neuron death [23] (Figure 1[F–H]). In this context, some authors hypothesize the cores of amyloid in the AD brain as a mechanism of defense, which in the end leads to catastrophic consequences [33]. 

### 1.2. Role of Cholinesterase Enzymes (ChEs) in AD

The first physiological evidence for the involvement of the cholinergic system in AD pathology was a reduction in pre-synaptic acetylcholine (ACh), and a reduced expression of the choline acetyltransferase (ChAT) enzyme responsible for ACh synthesis. According to the cholinergic theory, the development of AD symptoms is related to structural alterations in cholinergic synapses, loss of ACh receptors, death of ACh-generating neurons and the deterioration of cholinergic transmission (Figure 1[I–M]). Taken together, all these issues lead to the accumulation of the enzyme responsible for ACh hydrolysis, AChE and butyrylcholinesterase (BChE) (Figure 1[N]) [34,35]. Cholinergic neurotransmission is based on proteins involved in ACh synthesis, storage, transport, and degradation. Acetylcholine is synthesized from choline and active acetate partly in the cytoplasm of cholinergic neurons but mostly at the terminal buttons [36]. Choline originates from lipid degradation and it is captured from outside the neuron by axonal termination via a specific transport mechanism. Acetyl-coenzyme A (acetyl-CoA) is formed in the mitochondria starting from pyruvate. The esterification between choline and acetyl-CoA is catalyzed by ChAT, an enzyme present in high concentration in the cytoplasm of cholinergic nerve endings. The activity of ChAT is regulated by neuronal depolarization, influx of calcium ions and phosphorylation of the enzyme [36,37,38]. The release of acetylcholine occurs by exocytosis of synaptic vesicles. The vesicles fuse with the pre-synaptic membrane and eliminate the neurotransmitter in the synaptic cleft where it can activate two different types of receptor: muscarinic and nicotinic. The release activity is due to the influx of calcium ions, which occurs as a result of the opening of the slow channels in the pre-synaptic membrane, controlled by depolarization [38,39]. Acetylcholine crosses the synaptic cleft and enters with its cationic end in the anionic site of the active receptor surface and it is fixed with its ester group by the esterophilic site of the enzyme. Acetylcholine that breaks down from the cholinergic receptor complex is rapidly hydrolyzed and inactivated by AChE, an enzyme present in the synaptic cleft, either free or bound to the basal lamina. There are two types of cholinesterase, AChE and BChE. Both enzymes are α,β-hydrolases folded with an α-helix bound with β-sheet containing a catalytic domain [40]. Although AChE and BChE are structurally similar, both their significance and location are substantially different; AChE is predominantly observed in the neuronal synapses and blood, whereas BChE, at the level of the human brain, is located close to glial cells and neurons or in tangles and neuritic plaques in AD patients [41,42]. While through AD progression, AChE activity is gradually reduced, BChE activity slightly increases, thus both enzymes have drawn the attention of researchers as molecular targets for the design of dual AChE and BChE inhibitors in the interest of a better disease outcome [43]. 

In fact, current AD therapies aiming at the increase of ACh levels in the brain are able to target only AChE. Even though this pharmacotherapeutic approach leads to a partial stabilization of cognitive function and improvement of the quality of life, these compounds have beneficial effects only for a short period of time (usually 1–3 years) since they are not able to influence the disease evolution [44]. The active site gorge of both ChEs is deep ~20 Å length, wherein the catalytic site is located at the bottom of the gorge (∼4 Å above the base of gorge). Within the gorge, two distinct sites exist, the catalytic anionic site (CAS) and the peripheral anionic site (PAS) [45]. The active site includes a catalytic triad of aminoacidic residues Ser200, His440 and Glu327 that catalyzes the hydrolysis of the ester bond of the neurotransmitter and also an anionic site or α-anionic site which is characterized by a Trp84 residue, among other aromatic ones, that interacts with the quaternary ammonium of ACh, ensuring its correct orientation [46]. The catalytic mechanism is similar to that of other hydrolases where the hydroxyl group of the serine becomes highly nucleophilic by a charge-retransmission mechanism involving the carboxylate anion of glutamate, the imidazole anion of histidine, and the hydroxyl anion of the serine. During the enzymatic attack on acetylcholine, which is an ester with trigonal geometry, a tetrahedral intermediate is formed between the enzyme and the substrate [47]. The PAS site is located at the entrance of the gorge and is known to allosterically modulate the enzyme activity. Donepezil, a clinically approved drug for the treatment of AD, presents a structure which spans the entire active site, interacting with both CAS and PAS residues at the same time [39,48,49].

Interestingly, AChE peripheral anionic site has been reported to play an important role in AD pathogenesis since it contains a motif that promotes Aβ fibril formation (Figure 1[O]): the interaction of the Aβ peptide with the PAS contributes to the formation of amyloid plaques by accelerating the aggregation process. The PAS sequence responsible for triggering Aβ aggregation has been identified as a hydrophobic AChE sequence (aa 281–315) including Trp279 such as highly conserved key residue [50].

### 1.3. Role of Tau Protein in AD

Tau is a microtubule associated protein expressed primarily in neurons, which aggregates into neurofibrillary tangles (NFTs), one of the two pathological hallmarks of the disease along with the amyloid plaque deposits [51]. Tau is encoded by a single gene (microtubule associated protein Tau, MAPT) on chromosome 17, resulting in six isoforms in the central nervous system (CNS) and six additional isoforms in the peripheral nervous system (PNS) after alternative splicing. Tau protein presents four primary domains: the N-terminal domain, the proline-rich domain, the microtubule-binding domain (MBD), and the C-terminal region. Alternative splicing primarily affects the N-terminal and MBD, yielding 4-repeat (4R) and 3-repeat (3R) tau. These two isoforms are present in ratio 1:1 in adult human brains, and 4R tau demonstrates a stronger activity than 3R tau in inducing microtubule assembly. The disruption of the physiological ratio is at the base of several tauopathies, AD included. Several tau mutations have been observed and numbered by their locations in 2N4R human tau and are associated with the emergence of tauopathies. These mutations could impact tau post-translational modifications, protein folding and aggregation [52]. 

Intracellular tau aggregate formation is mediated by the MBD, in a region between Ser214 and Glu372 which binds microtubules tethering tubulin dimers together. MBD region contains a tau repeat domain (tau RD), which spans residues 243 to 365 [53]. The third repeat contains the hexapeptide motif ^306^VQIVYK^311^ which is the most important for fibril assembly since, along with a second hexapeptide motif ^275^VQIINK^280^, it promotes the formation of β-sheet structures and consequent tau aggregation. The occurrence of tau mutations (Figure 1[P]) that destabilize local structure around these motifs could trigger spontaneous aggregation (Figure 1[Q]), leading to tauopathies (i.e., missense mutations of Pro301 changed to Leu or Ser cause tauopathy and are associated with neurodegeneration in model systems) [52,53]. 

Tau phosphorylation and cleavage (Figure 1[R]) are two other subjects that need to be considered, since they represent the early steps that trigger and precede its aggregation. Tau contains 85 potential serine, threonine, and tyrosine phosphorylation sites, mainly found in the proline-rich domain of tau near the MBD. Tau hyperphosphorylation occurs in tauopathies: in normal brains, about 10 phosphorylated residues could be detected on soluble tau, while approximately 45 residues, representing more than 50% of all phosphorylable residues, have been found in AD brains. A large number of different kinases and phosphatases (Figure 1[S]) are involved in tau phosphorylation regulation, including glycogen synthase kinase-3 (GSK-3), cyclin-dependent kinase 5 (cdk5), and 50 adenosine monophosphate-activated protein kinase (AMPK), casein kinase-1(CK1), microtubule affinity-regulating kinases (MARKs), cyclic AMP-dependent protein kinase A (PKA), dual specificity tyrosine-phosphorylation-regulated kinase-1A (DYRK-1A), tyrosine kinases (Fyn, Abl and Syk) and phosphatases such as protein phosphatase-1, -2A, and -5 (PP1, PP2A, and PP5) [54].

Tau can be cleaved by many proteases: caspase-3 cleaves tau at Asp421 while calpain-1 and caspase-6 are responsible for the N-terminal cleavage. The resulting tau fragments have been detected in AD brains; in fact, caspase-cleaved tau fragments are known to be prone to aggregation, while cleavage of tau by calpain appears to partially inhibit the aggregation processes. Thus, phosphorylation and caspase-mediated cleavage of tau should be considered also as important events in triggering the NFTs formation in AD [53,54].

## 2. Quinone-Based Scaffolds for the Development of Novel Agents against AD

**Figure 2:**
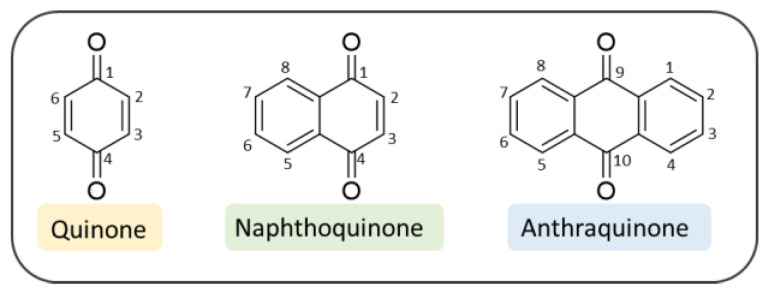
Structures of 1,4-quinone and related benzoquinones.

Quinones are interesting chemical structures whose main features include a non-aromatic ring and two carbonyl functions at the 1,4 or the 1,2 positions to one another. The three most common quinone-based derivatives are benzoquinones, NQs and AQs (Figure 2). In this review we will focus on 1,4-naphthoquinones (1,4-NQs) and 9,10-anthraquinones (9,10-AQs) that have been disclosed so far, during the search for valuable chemotypes with potential to treat AD.

## 3. Naphthoquinones

NQs are colored chemical compounds that exist in nature as secondary metabolites of plants that are used in many traditional medicines in Asian countries. NQs have gained considerable interest from researchers due to their antibacterial, antifungal, antitumor, and insecticidal properties [55,56,57,58].

The diverse set of pharmacological activities displayed by these compounds makes the NQ scaffold very attractive as a building block for drug development. The most stable isomeric form, 1,4-NQ, has been widely applied in organic reactions, such as Michael-type additions [59,60], aldol-type reactions, Diels-Alder reactions [61,62], cycloadditions [63], Friedel–Crafts reactions [64] and epoxidation [65,66] thanks to its two reactive functional groups, such as a C–C double bond and two ketone carbonyls.

Recent studies have also shed light on the neuroprotective effects and Aβ aggregation inhibition performed by 1,4-NQs [67,68,69,70], thus suggesting the NQ scaffold as valuable chemotype for the design of AD therapeutics. 

**Figure 3:**
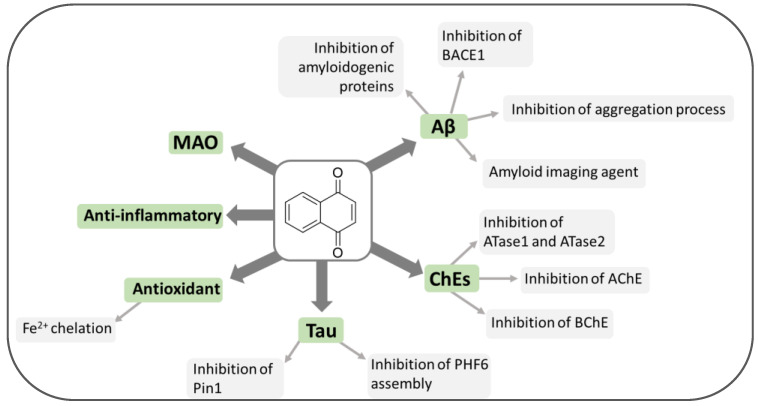
Biological profiles of 1,4-NQs in the treatment of Alzheimer’s disease.

NQ can be considered a privileged structure since its derivatives have demonstrated the ability to interact with several and different biological/pharmacological targets, thus exhibiting a wide range of activities. However, the effect of 1,4-NQs on neurodegenerative diseases has been subject to few studies. Only recently, natural and synthetic NQ derivatives have started to be explored as potential agents for the treatment of AD (Figure 3). 

### 3.1. NQs from Natural Sources

Since antiquity, the treatment and cure of human diseases with plant-derived extracts, powders, oils, roots, etc. have been widely recognized in medical practice. Natural products have been the source of valuable drugs for different pharmacological settings and have served as fragments for drug design strategy [71,72,73,74,75,76]. NQs represent a varied family of naturally occurring secondary metabolites [77,78,79,80], whose interest has intensified in recent years also for the treatment of AD.

**Figure 4:**
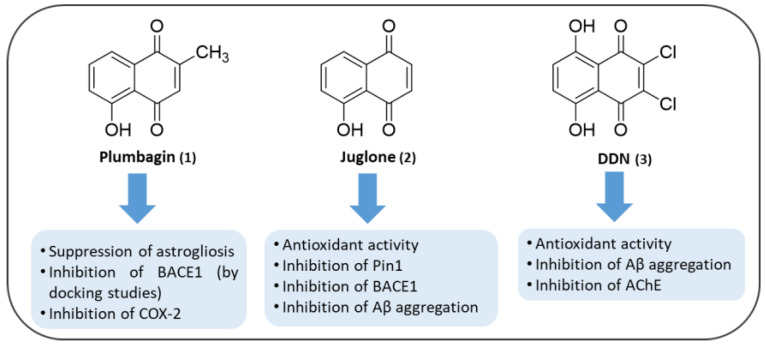
Chemical structures and biological properties of plumbagin, juglone and DDN against AD.

The first example is plumbagin (5-hydroxy-2-methyl-1,4-naphthoquinone (**1**), Figure 4), which is one of the simplest plant secondary metabolites of three major phylogenic families viz. *Plumbaginaceae*, *Droseraceae* and *Ebenceae* [81,82]. It exhibits potent biological activities, including antioxidant (by means of different assays) as well as prooxidant properties [83], as observed for other naturally occurring compounds [84,85] and also anti-inflammatory ones [86,87]. These properties suggested the activation of adaptive cellular stress response pathways as plausible neuroprotective effects [88]. This molecule has been tested by Nakhate et al. for its ameliorative effect on learning and memory in AD-like conditions in mice [3]. They treated mice with a daily intraperitoneal (i.p.) dose of plumbagin (0.5 and 1 mg/kg) starting from 1h prior to the first intracerebroventricularly treatment with streptozotocin (STZ; 3 mg/kg), a molecule able to recapitulate an AD-like condition. Plumbagin demonstrated the ability to prevent the loss of learning and memory in mice subjected to Morris water maze (MWM). They suggested that the anti-Alzheimer’s effect of plumbagin could be associated with activation of Nrf2/ARE signaling with consequential suppression of astrogliosis and inhibition of BACE1. They confirmed their hypothesis with the administration of a Nrf2/ARE inhibitor, trigonelline (10 and 15 mg/kg), which proved to enhance the effect of STZ. On the other hand, pre-treating mice with a sub-effective dose of trigonelline (5 mg/kg) attenuated the effect of plumbagin. Finally, docking studies allowed the demonstration of the excellent binding mode of plumbagin to B and D chains of BACE1 enzyme.

On this basis, plumbagin may deserve more in-depth studies in order to confirm its potential against AD [3].

Another interesting natural molecule is juglone (5-hydroxy-1,4-naphthoquinone (**2**), Figure 4), a phenolic compound produced by numerous species of walnut tree, found in the fresh ripe fruit husk, roots, leaves, and bark [89,90]. Ahmad et al. [91] reported that juglone demonstrated to have various pharmacological activities, including antimicrobial [92], anti-cancer [93,94,95], anti-fungal [92], antioxidant [96] as well as apoptotic [97] and anti-angiogenesis properties [98]. Juglone contains an intramolecular hydrogen bond between hydroxyl and keto groups and is active in donating the hydrogen-atom [99], thus it may have either pro- or anti-oxidant characteristics depending on the concentrations [96]. Accordingly, some studies have reported the generation of ROS by juglone, while others describe its antioxidant properties [100]. Furthermore, deprotonated juglone has demonstrated the ability to chelate Fe^2+^ [101] leading to the formation of stable complexes, thereby preventing this metal from participating in free radical generation [102,103,104,105], since ferrous iron promotes lipid oxidation through Fenton reaction [106]. Accumulating evidence suggest that antioxidant properties of juglone are useful in combating oxidative stress-linked diseases, being able to prevent oxidative and heat stress-induced dephosphorylation of Tau in human cortical neurons [107]. A recent study in a transgenic mouse model of AD demonstrated that the walnut supplement can reduce oxidative damage [108]. 

Juglone is also an inhibitor of Pin1, a parvulin member of peptidyl-prolyl cis/trans isomerases (PPIases) [109], that can regulate protein phosphorylation and cell signaling, catalyzing the cis/trans isomerization of peptide bonds preceding prolyl residues [110]. Pin1 inactivation occurs through a Michael addition of the thiol groups of Cys41 and Cys69 of the enzyme to two juglone molecules, forming covalent bonds [109]. Recently Pin1 activity has been connected to AD through the modulation of phosphorylation of Tau protein [107,111], hence Pin1 and juglone have gained considerable attention. Juglone has also been studied by Bescos et al. [70] for its ability to inhibit both BACE1 (IC_50_ = 6.51 µM) and the aggregation of β-amyloid (IC_50_ = 11.10 µM) and for its ability to disaggregate preformed amyloid fibrils (IC_50_ = 15.49 µM). Altogether, this information makes juglone a promising chemotype for the development of novel drugs for the treatment of AD.

The last example of natural NQ against AD is DDN (2,3-dichloro-5,8-dihydroxy-1,4-naphthoquinone (**3**), Figure 4), that has been tested by Khelifi et al. [112] for its antioxidant potential in two different assays. ABTS assay is based on the exchange of hydrogen atoms between the antioxidant and the stable radical [113], while iron reducing power test is based on the donation of a single electron transfer. The results showed that DDN has an excellent antioxidant activity with low IC_50_ (9.8 ± 0.2 μM) and EC_50_ (4.3 ± 1.6 μM) values for both tests, respectively, exceeding the potency values of ascorbic acid used as positive control. DDN has also been evaluated for its dual inhibitory effect on both Aβ_42_ aggregation and AChE. The results of ThT assay, for the inhibition of Aβ_42_, showed the formation of ThT positive species corresponding to the interaction of DDN with β-sheet structures of Aβ_42_ formed after 24 h. However, in the presence of 25, 50, and 100 μM of DDN, an almost complete reduction of ThT fluorescence was observed. The in vitro AChE inhibitory potency of DDN (IC_50_ = 14.5 ± 1.0 μM) was comparable to that of galantamine exibiting an IC_50_ equal to 9.3 ± 1.2 μM. Bermejo-Bescós et al. tested 5,8-dihydroxy-1,4-naphthoquinone in the same experimental conditions, without observing the inhibition of AChE [70]. This result has been related to the absence of 2,3-dichloro substitution in DDN. Thanks to molecular modeling studies, Khelifi et al. observed that DDN could share the same binding interaction (to Tyr337) of galantamine, used as reference compound [112].

Since quinones display low solubility in water and a limited stability, which impair their bioavailability, the authors also studied the drug releasing by encapsulation of DDN into alginate microspheres, in order to prevent enzymatic degradation and improve the blood-brain barrier (BBB) permeability. The compound release patterns suggested the release of 1040 μg/cm^2^ of the 25% diffused amount. Taking into account the first passage hepatic degradation after oral administration, they asserted that this quantity of DDN seems to be sufficient to ensure its therapeutic effectiveness. 

Other natural compounds demonstrated to have important biological activity of potential utility against AD, such as shikonin, which displayed anti monoamine oxidase (MAO) activity [114,115]. MAO enzymes have been identified as key contributors to AD pathogenesis, inducing the expression of β-secretase and γ-secretase with a subsequent increase in Aβ oligomerization and fibrillation [116]. Accordingly, MAO inhibitors are presently studied for their neuroprotective properties as new promising drugs for cognitive impairment in AD and other dementias. However, for shikonin and other natural products further studies are required with a view to connecting their beneficial effects to AD.

### 3.2. Synthetic NQ Derivatives

**Figure 5:**
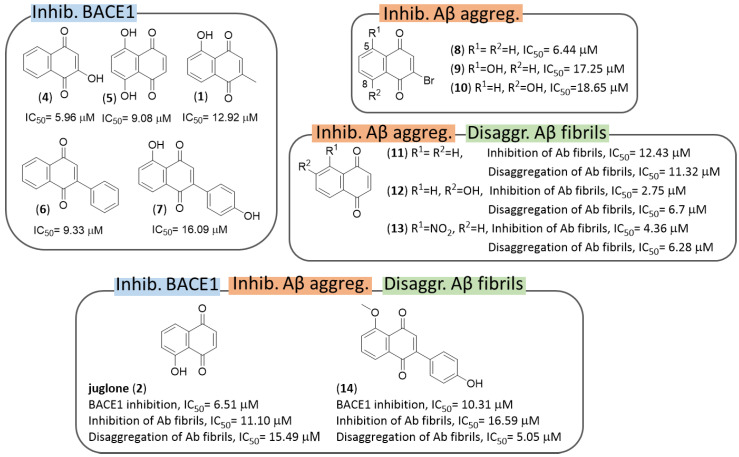
Anti-amyloidogenic properties of NQs investigated by P. Bermejo-Bescós et al.

As mentioned before, natural compounds may serve to select novel core structure and molecular fragments to enrich the molecular diversity of a compound library toward an enhanced efficacy. On this wave, Bermejo-Bescós et al. in 2010 screened 26 NQ derivatives, juglone being included, for their antiamyloidogenic properties [70]. Several 1,4-NQs provided activities in the inhibition of BACE1 and Aβ aggregation, and disaggregation of Aβ fibrils (Figure 5). In particular, five compounds (2-hydroxy-1,4-naphthoquinone (**4**), 5,8-dihydroxy-1,4-naphthoquinone (**5**), plumbagin (**1**), 2-phenyl-1,4-naphthoquinone (**6**) and 5-hydroxy-2-(4-hydroxyphenyl)-1,4-naphthoquinone (**7**)) displayed an inhibitory selective profile on BACE1, while 2-bromo-NQs behaved selectively as inhibitors of Aβ aggregation. 1,4-Naphthoquinone (**11**), 6-hydroxy-1,4-naphthoquinone (**12**) and 5-nitro-1,4-naphthoquinone (**13**) were dual inhibitors of Aβ aggregation and disaggregation. Finally, juglone (**2**) and 3-(4-hydroxyphenyl)-5-methoxy-1,4-naphthoquinone (**14**), showed a promiscuous pharmacological profile against all the three targets. 

Although these compounds have elicited toxicity at high doses, and some have been evaluated for their ability to arrest the cell growth or to kill cancer cells [96], at subtoxic doses they have proved to activate adaptive stress response pathways in neurons, protecting neurons against severe stress, thus being worthy of successive investigations as promising neuroprotective agents [88,117].

**Figure 6:**
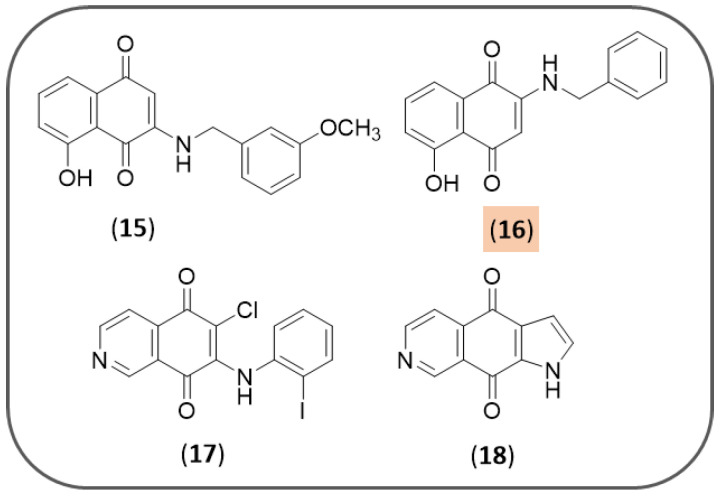
Synthetic NQ investigated by N. Neo Shin et al. as amyloid imaging agents.

Neo Shin et al. screened 41 1,4-NQ derivatives as inhibitors of Aβ aggregation (ThT assays) and 14 compounds were selected for further studies to check their ability to dissociate preformed Aβ aggregates [118]. However, conflicting results prompted the authors to further investigate the antiamyloidogenic properties of these derivatives. Docking and biophysical studies revealed that four compounds (**15–18**, Figure 6) are able to directly bind to amyloid-β aggregates and enhance their fluorescence properties in the presence with Aβ aggregates. These compounds specifically showed to stain both diffuse and dense-core amyloid-β plaques in brain sections of APP/PS1 double transgenic AD mouse models. 2-(Benzylamino)-5-hydroxynaphthoquinone (**16**) emerged as the best performing candidate, in virtue of its ability to enhance fluorescence by 50-fold when its emission is collected from 680 to 750 nm. Altogether, this study has aroused interest for 1,4-NQ-based molecules to serve as amyloid imaging agents for diagnosing early AD patients.

**Figure 7:**
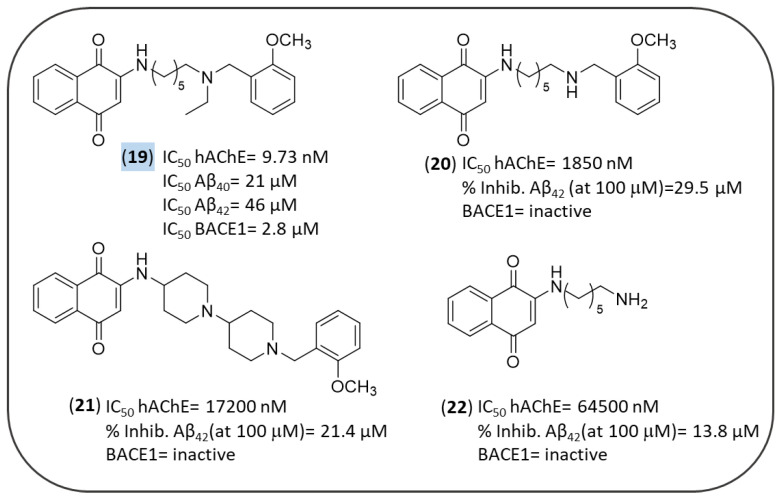
NQs studied by M.L. Bolognesi et al.

In 2011, Bolognesi et al. designed several derivatives of the bivalent ligand memoquin in order to simplify its structure and reduce its MW, while still preserving its multitarget profile [119]. Among them, four NQs (**19**–**22** of Figure 7) were evaluated against multiple AD targets such as AChE, self-induced Aβ aggregation, and BACE1 (Figure 7).

As a result of AChE inhibition study, only compound **19** proved to be effective with IC_50_ = 9.73 nM. The lower activity of **20** and **22** confirmed both the ethyl group and the 2-methoxybenzyl moiety as important substitutions of the terminal tertiary amine group, as previously observed in other memoquin derivatives [120,121]. Conversely, the 1,4-piperidine spacer of **21** was too rigid, thus hampering its adequate fitting into the AchE gorge. Docking simulations showed that **20** was able to interact with the *h*AChE catalytic site, and meantime to protrude towards the solvent-exposed gorge entrance, establishing three key interactions at *h*AChE active site: (i) protonated nitrogen of the ligand formed a cation−π interaction with the indole ring of Trp86 and the phenol ring of Tyr337; (ii) the oxygen in position 1 of the quinone moiety made a H-bond with the backbone of Phe295; (iii) the NQ moiety was engaged in favorable π−π stackings with the indole ring of Trp286 of the PAS. The last finding was relevant in the context of previous reports which connected the inhibition of AChE-induced Aβ aggregation to the ability of a binder to interact with the PAS of the enzyme. Hence, a direct correlation between AChE inhibition and AChE-induced aggregation was observed for **20** [122,123]. Indeed, the inhibition of self-induced Aβ aggregation by **19** was lower than **21** (22% vs. 29%, respectively) probably because compound **21** could take additional positive contacts with the biological target through its additional nitrogen atom. Compound **19** was tested in primary chicken telencephalon neurons to substantiate its secretase inhibitory activity by affecting APP processing [124]. Interestingly, **19** inhibited Aβ_38_, Aβ_40_, and Aβ_42_ secretion, with IC_50_ values of 19, 21, and 46 μM, respectively, without producing toxic effects in a concentration range of 0.01−50 μM.

**Figure 8:**
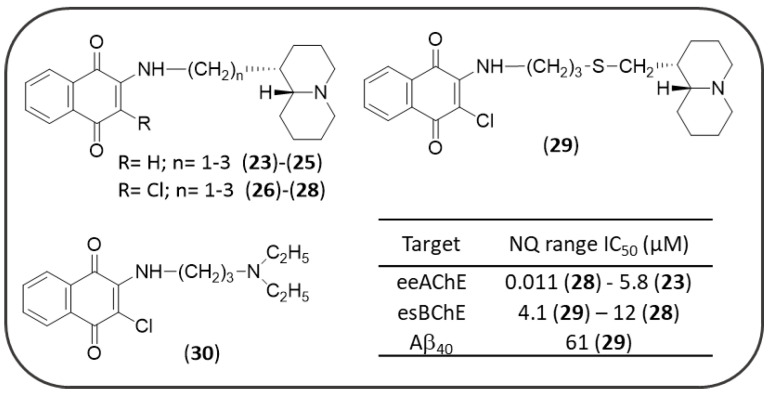
NQ derivatives studied by Sparatore et al. as ChEs and Aβ aggregation inhibitors.

In 2015, Sparatore F. et al. described the multitarget profile of a library of thioxanthene-9-one, xanthen-9-one, NQ (Figure 8) and AQ derivatives (see Figure 18) decorated with a basic side chain of variable length (dialkylaminoalkyl and quinolizidinylalkyl chains) [125]. These molecules were tested against electric eel AChE (*ee*AChE) and equine serum BChE (*es*BChE) and the spontaneous Aβ_40_ aggregation. In particular, most of NQs **23–30** proved to be dual but AChE-preferring inhibitors (IC_50_ = 0.011–5.8 µM) over BChE, while Aβ_40_ aggregation was poorly inhibited (**29**, IC_50_ = 61 µM) or not affected. Regarding the influence of the polymethylene linker tethering the NQ scaffold to the basic moiety, its elongation was responsible for an increase of the ChE inhibitory potencies. However, a remarkable 37-fold decrease of AChE inhibition was observed when the trimethylene linker of **28** (IC_50_ = 0.011 µM) was further elongated of two units (**29**, IC_50_ = 0.41 µM). Compound **28** confirmed the same degree of activity against the human AChE (*h*AChE, IC_50_ = 0.04 µM). As lead of NQ subset, it was also investigated for its ability to cross the BBB by passive diffusion, βand to interact with P-glycoprotein (P-gp), which is involved in the efflux transport of drugs: it showed an efflux ratio (ER) equal to 0.78 comparable to that of diazepam (ER = 0.79), used as reference compound. In vitro assay for cytotoxicity against the neuroblastoma cell line SH-SY5Y revealed for compound **28** a low IC_50_ value of 3.6 µM, but a good selectivity ratio (toxicity/AChE inhibition) equal to 327.

The mechanisms that regulate levels and activity of BACE1 may serve for therapeutic purpose of AD. Nascent BACE1 to complete maturation has to be transiently acetylated by two endoplasmic reticulum acetyl-CoA:lysine acetyltransferases, named ATase1 and ATase2 [126] (Figure 3). These enzymes are up-regulated in the brain of AD patients and increase the levels of BACE1 and the generation of Aβ. Interestingly, from a step-by-step screening of a library of 14,400 compounds, Puglielli L. et al. identified the 2-chloro-3-(2-ethoxyanilino)-1,4-naphthoquinone and a phenoxazin-5-one derivative as promising compounds, being able to selectively down-regulate ATase1 and ATase2 activity in vitro, without interfering with the acetylation of other classes of proteins [127].

### 3.3. NQ-Based Hybrids

The simplest way to incorporate two (or more) different activities in one single molecule is the combination of their respective pharmacophoric elements, that are responsible for the diverse biological properties [128]. The multitarget approach may be considered an evolution of this concept. The rationale for using the bivalent ligand approach stems from the possibility of tackling the intricate array of AD, through dimeric structures capable of bridging independent recognition sites of validated targets (such as AChE, Aβ and BACE1).

**Figure 9:**
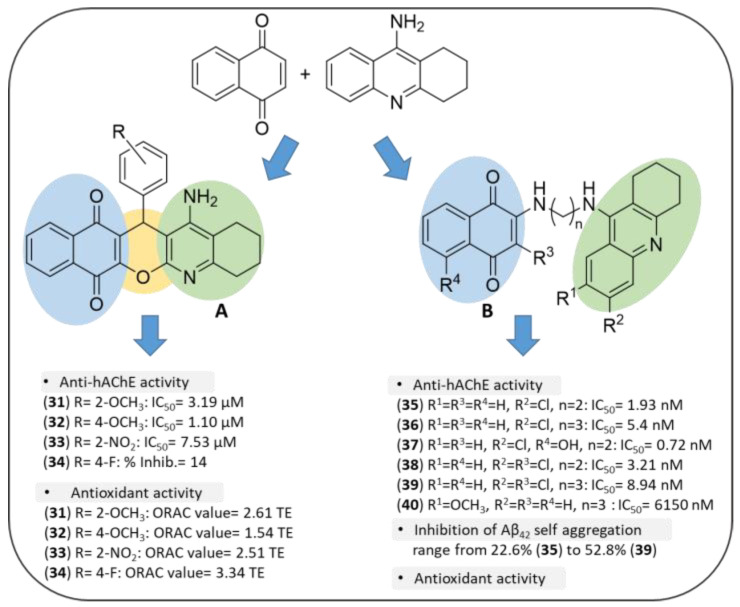
NQ-tacrine hybrids as MTDLs. QuinoPyranTacrines (**A**) and NQ-tacrine hybrids (**B**).

**Figure 10:**
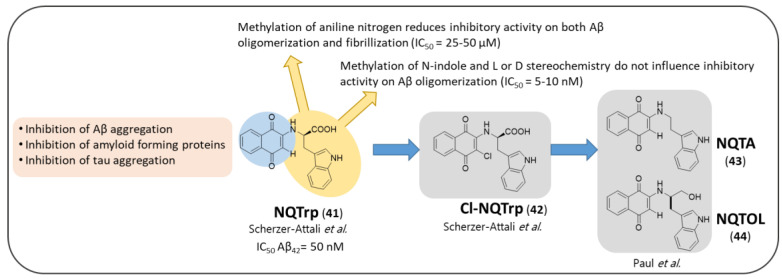
NQ-tryptophan (NQTrp) hybrids against AD.

In this context, the NQ scaffold was included in three different types of hybrid molecules (Figure 9 and Figure 10). In two of them, the NQ nucleus was combined with the structure of tacrine, which was the first marketed AChE inhibitor for AD therapy, and withdrawn from use in 2013 due to its hepatotoxicity [129]. Despite this, tacrine continues to be used as a template for the design of new safer analogues against AD [130]. 

QuinoPyranTacrines (QPT), by M. Chioua et al., resulted from the condensation of 1,4-NQ and tacrine motifs (**31**–**34**, Figure 9A) by means of a 4*H*-pyran ring, bearing at position 4 an aromatic ring variously decorated with electron-withdrawing (F, NO_2_) and electron-donor (OCH_3_) groups [131]. In the initial screening phase, all the compounds were tested for their potential hepatotoxicity, resulting non-hepatotoxic in the majority of cases. The evaluation of their inhibitory action against human ChEs allowed only three compounds to be identified as hAChE inhibitors. The 4-methoxy substituted derivative (**32**) was the most active, displaying IC_50_ = 1.10 μM, but less potent than tacrine, and inactive against BChE. The compounds were next evaluated for their antioxidant activity and shared to scavenge the peroxyl radical with ORAC (oxygen radical absorbance capacity) values in the range 1.54 (**32**)–3.34 (**34**) Trolox equivalents (TE), comparable to reference compound ferulic acid (ORAC value = 3.74 TE). In particular, the electron withdrawing groups such as R *=* 4-F (**34**) and 4-NO_2_ (**33**) were responsible for a greater antioxidant activity, while for OCH_3_ group the substitution at position 2 of the aromatic ring was preferred (**31**). Docking studies allowed the disclosure of the R-enantiomer of **32** as the best conformation to fit into the AChE cavity. Thus, compound **32** may represent an interesting starting point for the design of novel tacrine-based hybrids with enhanced efficacy and safety. 

Another example of NQ-tacrine hybrids was proposed by E. Nepovimova et al. who connected the two scaffolds through a polimethylene chain (Figure 9B) with the aim to target simultaneously Aβ aggregation, AChE and oxidative stress [132]. 1,4-NQ, 2,3-dichloro-1,4-NQ and 5-hydroxy-1,4-NQ (juglone) were selected as scaffolds to be joined to 6-chlorotacrine, which had showed an improved AChE inhibitory profile with respect to tacrine [133], and to its 7-methoxy analogue, which confirmed a comparable efficacy against AChE, but with significantly lower side effects compared to the parent drug, probably due to a different metabolic fate [134]. The length of the linker connecting the two frameworks was set according indications derived from preliminary docking simulations, that suggested two or three methylene units as the best suited distance for a comfortable positioning of the hybrid’s subunit within the AChE gorge.

All the hybrids turned out to be effective inhibitors of *h*AChE [132], in a wide range of IC_50_ values from micromolar to sub-nanomolar concentrations (Figure 9B), surpassing the potency of tacrine (IC_50_ = 500 nM). The presence of tacrine moiety undoubtedly contributed to their inhibitory activity. Both the highest potency against AChE (sub-nanomolar to one-digit nanomolar, Figure 9B) and the selectivity over BChE were displayed by 6-chlorotacrine derivatives (R^2^ = Cl, **35–39**). The unsubstituted tacrine derivatives showed moderate activity (hAChE IC_50_ = 38–53.7 nM), while the 7-methoxytacrine compounds (**40**, as an example) had dramatically lower potencies falling in the sub-micromolar or micromolar range (*h*AChE IC_50_ = 348–6150 (**40**) nM). The best result was obtained by the 6-chlorotacrine derivative of juglone which exhibited an IC_50_ = 0.72 nM (**37**).

In general, structural modifications of tacrine scaffold led to a drop in the inhibitory activity on BChE except for the tacrine-1,4-NQ hybrid, which was 4-fold more potent than tacrine. 7-Methoxytacrine-1,4-NQ-based derivative (**40**) was the only BChE preferential inhibitor (*h*BChE IC_50_/*h*AChE IC_50_ = 10) in the series. Analysis of X-ray crystal structure of the complex between *Torpedo californica* AChE (*Tc*AChE) and the best AChE inhibitor (6-chlorotacrine—juglone hybrid) showed that the 6-Cl atom interacted with the CAS via hydrophobic contacts, the methylene chain was involved in water-mediated hydrogen-bonds and the juglone moiety accommodated in the narrow bottleneck of AChE, making van der Waals interactions [132]. In general, hybrids characterized by a propylene chain as spacer were more potent than those endowed with ethylene one, whereas for juglone derivatives, carrying the 5-OH group, likely involved in a hydrogen bond, even a shorter link was permitted, allowing the molecule to properly fit within the AChE cavity. The molecules were also tested against spontaneous amyloid aggregation at 10 μM, and 2-chloroquinone scaffold linked to 6-chlorotacrine resulted to be the best suited for the activity (**39**, Inhib. Aβ aggreg. % = 52.8), whilst the unsubstituted and the 7-methoxy tacrine-based inhibitors resulted to be less effective (Inhib. Aβ aggreg. % = 20–30). Then, the authors verified the neurotoxic profile of the compounds in immortalized mouse cortical neurons Neuro2A (N2A) and primary rat cerebellar granule neurons, observing that most of them showed no significant reduction in cell viability compared to untreated cells. Hence, the two best performing molecules, the 6-chlorotacrine derivatives of 1,4-NQ (**35**) and juglone (**37**), were evaluated for their neuroprotective activity against two different harmful stimuli, such as Aβ_42_ and oxidative stress. The cell viability significantly increased in N2A cells pre-incubated with the two compounds at 12.5 μM, and then treated with Aβ_42_ peptide, compared to the cells only incubated with Aβ [132].

The antioxidant properties of the most active compounds were tested through the evaluation of ROS scavenging effects against human glioma T67 cells exposed to high level of tert-butyl hydroperoxide (TBH, 100 μM) in the presence or absence of pre-treatment with sulforaphane, used as a potent inducer of NAD(P)H quinone oxidoreductase 1 (NQO1), which is an inducible enzyme involved in the conversion of quinones into the more antioxidant hydroquinone forms [132]. Remarkably, the treatment with the juglone derivative of 6-chlorotacrine (**37**) completely suppressed TBH-induced intracellular ROS production, confirming the expected antioxidant properties of this compound. Importantly, *ex-vivo* experiments revealed the capacity of these hybrids of permeating the BBB, a fundamental requirement to access to their multiple biological targets in the CNS.

Finally, the last example of hybrid-based strategy was proposed by Scherzer-Attali et al. who in 2010 designed and synthesized a small library of NQ-tryptophan hybrid molecules (Figure 10) as candidate inhibitors of amyloid assembly [67]. These compounds demonstrated their efficacy by inhibiting β-amyloid aggregation in in vitro, in silico and in vivo experiments. The idea of conjugating the NQ and tryptophan moieties aimed to combine the crucial role of tryptophan during the amyloidogenic process [135] and the recognized ability of quinones to impair amyloid aggregation. Among the compounds tested, the N-(1,4-naphthoquinon-2-yl)-L-tryptophan (NQTrp, **41**) hybrid was found to be the most effective against Aβ_40_ aggregation, even at low molar ratios of 4:1 (Aβ_40_:NQTrp). A similar experiment with Aβ_42_ resulted in an IC_50_ = 50 nM (Figure 10). Transmission Electron Microscopy (TEM) imaging and circular dichroism (CD) spectroscopy showed a drastic fibrils reduction and a decrease in the β-sheet conformation, respectively. The affinity constant of NQTrp toward early oligomers of Aβ_42_ was estimated to be 90 nM. Additionally, the authors assessed the effect of NQTrp on Aβ oligomers using transgenic *Drosophila melanogaster* expressing human Aβ_42_ as animal model. The flies were fed with NQTrp throughout their lifespan; notably, the treatment prolonged their lifespan and completely abolished their defective locomotion. The western blot analysis of the fly brains showed an important reduction of Aβ oligomeric species. They also tried to elucidate the mechanism of interaction between NQTrp and Aβ peptides using Nuclear Magnetic Resonance (NMR) spectroscopy and Molecular Dynamics (MD) simulations observing a greater interaction with the central aromatic core of Aβ forming hydrogen bonds with its Phe20-Glu22 region. In 2014, Zhang et al. [136] demonstrated via MD simulation that the interaction is very dynamic and multiple, and evolves through many transient binding contacts; hence, in addition to the central hydrophobic core (residues 17–21) and the side chains of Phe19 and Phe20, the hydrophobic residues Leu34/Met35 and hydrophilic/polar residues Arg5, Asp7, Tyr10, His13, Lys16, and Lys18 were identified as hot spots for NQTrp binding to Aβ_42_. 

Scherzer-Attali et al. tested an analog of NQTrp, named Cl-NQTrp (**42**, Figure 10), for its effect on in vitro Aβ aggregation and in vivo accumulation [137]. Cl-NQTrp was able to completely inhibit the fibrillization and oligomerization of Aβ in vitro as well as to extend the lifespan and to improve the defected locomotive behavior of transgenic *Drosophila melanogaster.* Furthermore, Cl-NQTrp was found to correct cognitive defects in a severe AD mouse model, markedly decreasing oligomerization and Aβ plaques load in their brains. 

In the same year, NQTrp and Cl-NQTrp were tested against different amyloid forming proteins and peptides, both neuronal as α-sinuclein, and non-neuronal such as Islet Amyloid Polypeptide, Prostatic Acid Phosphatase Peptide (PAP), calcitonin, insulin and lysozyme [138]. 

Successively, Frenkel-Pinter et al. tested NQTrp and Cl-NQTrp as inhibitors of tau aggregation both in vitro and in vivo [139,140] by using a paired helical filament, PHF6 (highly repeated sequence ^306^VQIVYK^311^ responsible for aggregation of tau in paired helical fragments (PHF) [141], as an in vitro model. Tau aggregates are formed by the self-assembly of misfolded tau protein monomers into harmful oligomers and abnormal fibers called paired helical filaments (PHFs) that form higher-order β-sheet rich aggregates termed neurofibrillary tangles. PHFs consist of two filaments twisted around one another with a width of 8–20 nm and a cross-β-sheet conformation [142]. Thus, the authors estimated the potency of the two compounds to inhibit PHF6 aggregation by different biophysical techniques [139,140]. Both NQTrp and Cl-NQTrp were found to inhibit PHF6 aggregation in a dose-dependent manner, obtaining the maximum inhibition at 1:5 molar ratio (PHF6: NQTrp/Cl-NQTrp). The same behavior was further validated by CD spectroscopy and TEM imaging. Then they examined the in vivo efficacy using transgenic *Drosophila melanogaster* overexpressing human Tau in its central nervous system or in its retina. The treatment with either NQTrp or Cl-NQTrp reduced the accumulation of Tau and its hyperphosphorylation, extended fly lifespan and generally led to an amelioration of tauopathy-related defects compared to the untreated flies. Both compounds disassembled preformed PHF6 fibrils in a dose-dependent manner with a maximum reduction of 40% obtained by 1:5 molar ratio (PHF6: NQTrp/Cl-NQTrp). MD simulation elucidated the interaction between NQTrp and Cl-NQTrp to PHF6 which exploited the same pattern of hydrogen bonds and π-π stacking, in line with the results for Aβ discussed above. It remains to be seen whether the results can be replicated in a rodent tauopathy model [139,140].

On this wave, Scherzer-Attali group designed and synthesized four derivatives of NQTrp (Figure 10) exploring the impact of configuration change (D-isomer of Trp) and of single and double-methylation of NQTrp nitrogen atoms on affecting Aβ aggregation [143]. The effects of the different substitutions and rearrangements were studied in silico as well as in vitro. The in-silico results suggested that the D-isomer and the N-methylindole derivative had a binding affinity toward Aβ oligomers comparable to NQTrp, while the N-methylnaphthoquinone and the dimethyl derivative were less efficient inhibitors and have lower affinity. Therefore, methylation of the indole nitrogen, as well as L or D stereochemistry did not seem to influence the inhibitory activity of Aβ oligomerization (IC_50_ = 5–10 nM), nor the affinity (K_d_ = 90 nM) versus Aβ oligomers. However, regarding the inhibitory activity on Aβ fibrillization, a different effect was observed. The N-methyl indole derivative displayed a reduced inhibition of fibrillization (IC_50_ = 50 μM), which was tentatively ascribed to a lower solubility, according to its more hydrophobic nature compared to NQTrp. On the other hand, as expected, the methylation of aniline nitrogen reduced the inhibition toward both fibrilization (IC_50_ = 25–50 μM) and oligomerization of Aβ, demonstrating that the hydrogen bond formed between Aβ and the aniline position of NQTrp is crucial both for binding and for inhibiting the aggregation. Regarding the double-methylated derivative, the authors did not present experimental data, due to the unstable nature of the compound, but the in-silico results were in line with that reported above. 

Another attempt of NQTrp optimization was made by Paul et al. in 2019, who designed and synthesized NQTrp analogs (Figure 10) with the NQ moiety linked with covalent bond to tryptamine (NQTA, **43**) or L-tryptophanol (NQTOL, **44**) [144]. These analogs were evaluated for the inhibition of aggregation of amyloids and disaggregation of preformed fibrillar assemblies of PHF6, Aβ_42_, and *h*IAPP in vitro. The hybrid molecules appeared to be more efficient modulators toward the slowly aggregating peptides (Aβ_42_ and *h*IAPP) than the fast-aggregating peptide (PHF6). This is probably due to the slow rate of primary nucleation of peptide molecules, which gives an adequate time for interaction of the inhibitor molecules [145,146]. These hybrids were also found to be non-toxic toward the neuroblastoma (SH-SY5Y) and kidney (HEK-293) cell lines and ameliorated the cytotoxicity induced by PHF6, Aβ_42_, and *h*IAPP aggregates. Molecular docking studies revealed that the hybrid molecules displayed significant interactions with the peptide monomers facilitating the inhibition of aggregation. In agreement with previous reports [67,147,148,149,150,151,152,153], the study revealed that the hybrid molecules interacted with various residues of Aβ fragment including Glu11, Val12, His13, His14, and Lys16, in addition to Leu17, Val18 located in the core hydrophobic region (^16^KLVFF^20^) [154], and this possibly rendered their inhibitory effects toward Aβ_42_ aggregation. To further validate the results of the Aβ fragment, the authors also performed a docking study of the hybrid molecules with Aβ_40_, and the predicted binding energies between them were in a similar pattern as with the Aβ fragment. They observed that the hybrid molecules interacted with the different residues of Aβ_40_ peptide, namely His6, Asp7, Ser8, His13, Gln15, Lys16, and Val18 through hydrogen bonding and hydrophobic interactions, which were also very similar to those observed during Aβ aggregation and agreed with previous reports [152,153]. Additionally, molecular dynamics simulation provided a plausible mechanism for disassembly of preformed fibrils arbitrated by the hybrid molecules. Namely, the hybrid molecules form hydrogen bonds predominantly with Val residue of PHF6, and Val was found to be the key residue in maintaining the β-sheet conformation between the two PHF6 peptide pairs. Thus, these researchers hypothesized that the interaction of the hybrid molecules with the hydrogen bond forming residues of PHF6 peptide might disrupt the existing peptide-peptide interaction in the β-sheet rich fibrillar arrangement, eventually disassembling the preformed aggregates. It is noteworthy that in all in vitro assays as well as in the computational studies, NQTOL appeared to be superior of the other hybrid molecules tested up to now. Collectively, these results strongly support the anti-amyloidogenic potential of NQTrp for the development of novel therapeutics against AD and other proteinopathies.

## 4. Anthraquinones 

**Figure 11:**
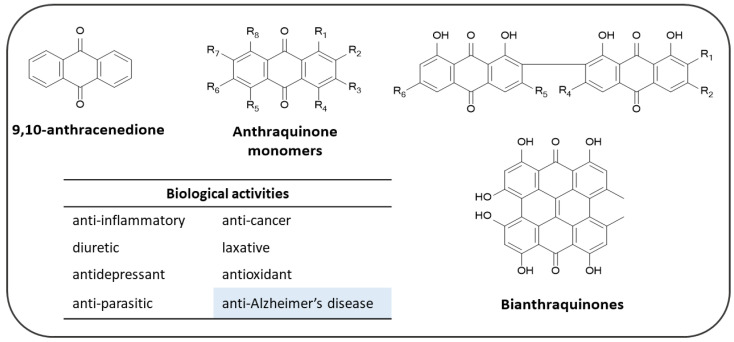
Overview of the biological properties of 9,10-anthracenedione parent scaffold and of the related derivatives from natural sources.

Among the AQ-based molecules which have been and are being currently studied as potential central nervous system (CNS) active agents, most of them derive from natural sources. To date, more than 700 natural AQs have been isolated from plants, lichens and fungi. In plants, AQ metabolites are present in a wide range of species, predominantly in the families of *Rubiaceae*, *Polygonaceae*, and *Rhamnaceae*. These compounds are structurally derived from the 9,10-anthracenedione nucleus [155], that is present in the form of monomers and bi-AQs, when including in their structures one or two basic cores, respectively [156] (Figure 11).

In the field of drug discovery, the AQ nucleus is an important scaffold associated with a wide range of pharmacological properties including anti-inflammatory, anti-cancer, diuretic, laxative, antidepressant, antioxidant and anti-parasitic activities (Figure 11) [157]. 

Recently, the class of natural AQ compounds has aroused great interest for its ability of hitting different molecular targets involved in AD (Figure 11). Different AQ-based molecules have shown to be capable of reducing the loss of cholinergic function in Alzheimer’s patients by acting as cholinesterase inhibitors [49], reducing the formation of protein aggregates, or acting as antioxidants, thus impairing the increased ROS formation associated with the AD progression.

### 4.1. AQs from Natural Sources: Cholinesterase Inhibitors

AQs extracted from different botanical sources have been studied and tested against human AChE and BChE. The chemical composition of the purified plant extracts of many species such as *Rumex abyssinicus*, *Cassia senna*, *Cassia occidentalis*, *Rheum palmatum L.*, *Aloe vera*, *Polygonum multiflorum* etc. has been determined and several 1,8-dihydroxyanthraquinone-based compounds have been isolated and screened for a potential application in AD.

**Figure 12:**
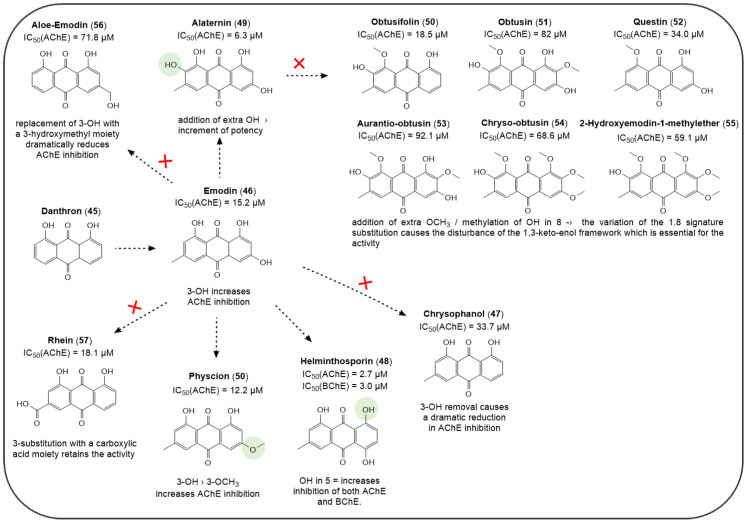
Representative AQs as ChEs inhibitors; x symbol indicates the negative impact on the activity exerted by some substitutions in a few emodin analogues.

Many derivatives of danthron (**45**, the unsubstituted 1,8-dihydroxyanthraquinone), have been evaluated in different studies for their inhibition of AChE and BChE, advocating their potential application for AD treatment. While danthron showed no activity against ChEs in vitro, its analogue, emodin (**46**, 6-methyl-1,3,8-trihydroxyanthraquinone), which can be found as a major constituent in several plants extracts, such as *Rheum Palmatum L., Rheum Abyssinicus, Cassia Obsutifolia* etc., has been frequently reported in the literature for its anti-AChE activity [49,158] (Figure 12). The presence of extra 3-hydroxyl- and a 6-methyl- groups on the danthron scaffold provided a selective anti-AChE profile (emodin, IC_50_ (AChE) = 9.17 ± 0.41 μM [158]; 15.21 ± 3.52 μM [49]) over BChE (emodin, IC_50_ (BChE) = 157 ± 2.03 μM [158]). Notably, the importance of the 3-hydroxyl group to afford AChE inhibition was observed in similar natural 1,8-dihydroxyanthraquinones (Figure 12). Chrysophanol (**47**), whose structure differs from emodin due to the absence of the 3-hydroxyl key group, has been reported to be at least ~2 fold less effective AChE inhibitor (IC_50_ = 68.6 ± 0.84 μM [158]; 33.7 ± 1.83 μM [49]).

In a recent paper, Augustin et al. characterized the *Rheum abyssinicus* extract describing the anti-cholinesterase activity of one of its AQ components, helminthosporin (**48**, 3-methyl-1,5,8-trihydroxyanthraquinone) against *ee*AChE and *es*BChE enzymes. This compound was a more potent AChE inhibitor than emodin (IC_50_ = 2.63 ± 0.09 μM [49]), and showed to target BChE with the same degree of potency (IC_50_ = 2.99 ± 0.55 μM [49]). For this reason, helminthosporin resulted an interesting hit as dual acting ChEs inhibitor. It is worth nothing how the different position of the 5-OH group of helminthosporin, with respect of the 3-OH group of emodin, was responsible of a ~5-fold increase of AChE inhibition and for the appearance of anti-BChE action. Molecular docking studies of helminthosporin in complex with the AChE enzyme have been performed, showing that the presence of an additional keto−enol tautomer (C_5_−OH, C_10_−CO) is responsible for the establishment of H-bond interactions with Arg296, Ser293 and Phe295 residues in the PAS site of the enzyme. Thus, these additional contacts could be accounted for the better activity of helminthosporin over other AQs [49].

Jung et al. also reported the anti-cholinesterase activity of the soluble extracts of *Cassia Obsutifoliae* and, after isolation of their components, they observed that the best inhibitors were 1,8-dihydroxyanthraquinones such as alaternin (**49**), physcion (**50**) and emodin (**46**) which displayed IC_50_ values versus the *h*AChE ranging from 6.3 to 15.2 μM [158]. Alaternin also exhibited such degree of activity towards AChE, with a modest inhibition of BChE (IC_50_ = 113 μM). Conversely, the 8-methoxy- or 1,8-dimethoxyanthraquinone analogues, such as obtusifolin (**50**), obtusin (**51**), questin (**52**), aurantio-obtusin (**53**), chryso-obtusin (**54**), 2-hydroxyemodin-1-methylether (**55**) were inactive or significantly less effective (Figure 12) [158]. Interestingly, the insertion of a hydroxymethyl chain or of a carboxylic function in place of emodin 3-OH group led to a reduced AChE inhibition, as experienced by aloe-emodin (**56**, IC_50_ (AChE) = 71.8 ± 0.91 μM [49]; IC_50_ (AChE) = 57.2 ± 1.32 μM [159]) and rhein (**57**, IC_50_ (AChE) = 18.1 ± 0.24 μM [159]).

**Figure 13:**
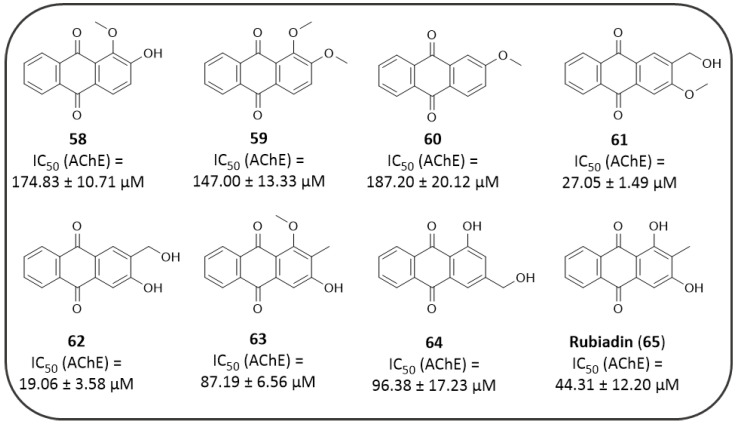
Other examples of AQs, devoid of 1,8-dihydroxy signature.

The relevance of the 1,8-dihydroxy substitution for an efficient AChE inhibition is also supported by the anti-cholinesterase activity elicited by the purified extracts of *Morinda Officinalis*, a tropical plant member of the *Rubiaceae* family. This plant is largely used in Chinese traditional medicine for the treatment of various diseases, and its extracts were found to be endowed with various biological activities, such as, inter alia, antioxidant, anti-inflammatory and anti-AD activities [160]. The *M. officinalis* ethyl acetate and hexane fractions were obtained by Lee Y. et al. who identified eight AQ compounds (**59–65**) lacking the 1,8-dihydroxy signature substitution (Figure 13) [161].

Accordingly, the novel chemotypes exhibited a lower activity profile than the previously discussed natural analogues (see Figure 12). In this set of AQs the SAR analysis revealed that the 1-methoxy substituent (i.e., **13–15**, **18**) clearly causes a dramatical decrease of anti-AChE activity (*ee*AChE) in comparison to the unsubstituted or 1-hydroxy substituted series (i.e., **59**, **62**, **64** and **65**) [161]. Notably, the 2-methoxy group seems to be not favourable for the activity (i.e., **59** and **60**); this trend may also be observed by comparing the activities of obtusine and aurantio-obtusine with those of questine and obtusifoline (Figure 12). Differently, the 2-carbinol substitution combined with 3-hydroxy or methoxy one (i.e., **61** and **62**) led to the most potent compounds. Position 3 permitted a certain chemical variation of substituents, which impaired the activity less drastically; even so, comparing the two most active compounds of this series, the replacement of the 3-methoxy group with the 3-hydroxy one enhanced the AChE inhibitory potency, and this is suggestive of a better influence of a less bulk and/or HB donor group in this position. These derivatives resulted selective AChE inhibitors, with IC_50_ > 200 μM against *es*BChE enzyme [161]. These compounds, and some of aforementioned AQs have been also tested as BACE1 inhibitors, whose activities will be discussed in a forthcoming paragraph.

### 4.2. AQs from Natural Sources: Tau Aggregation Inhibitors

Tau protein is an unfolded brain protein involved in the axonal transport associated with microtubules. In neurodegenerative diseases, such as AD, tau protein is hyperphosphorylated in vivo. This promotes tau detachment from microtubules and auto-aggregation forming toxic oligomers, which cause an inflammatory response.

**Figure 14:**
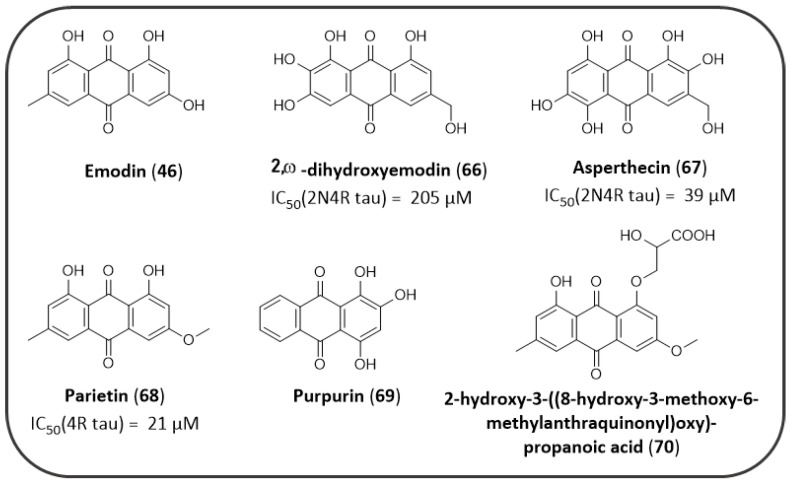
Natural derived AQs as tau aggregation inhibitors.

Several natural derived AQs (Figure 14) have been screened to evaluate their neuroprotective properties as inhibitors of tau aggregation. Emodin (**46**), which has been demonstrated to act as a good AChE inhibitor, is known to act as a tau oligomerization inhibitor as well [162,163]. Pickhardt et al. demonstrated for the first time the ability of emodin to block the in vitro polymerization of tau protein K19 (three-repeat tau construct), after heparin stimulation (IC_50_ values for PHF polymer assembly and disassembly = 1.6 μM and 2.8 μM, respectively) [162]. On the other hand, Paranjape et al. observed that emodin did not show a significant inhibitory activity against tau aggregation. This discrepancy was attributed to the use of arachidonic acid in place of heparin as tau aggregation inducer, able to induce a 3-fold superior amount of aggregates in similar experimental conditions. In addition, the two research groups used two different tau isoforms that could be featured by a slightly different aggregation behaviour. In the same study, two 1,8-dihydroxyanthraquinone compounds were obtained from genetic manipulated *A. nidulans*, which showed an improved profile: 2,ω-dihydroxyemodin (**66**) and asperthecin (**67**) caused a decrease in tau filament formation (IC_50_ values of 205 ± 28 µM and 39 ± 2 µM, respectively) in a dose dependent manner, while still retaining the physiological tau function of stabilizing the assembly of tubulin into microtubules [163]. Other AQ compounds (i.e., chrysophanol, aloe-emodin, endocrocin, ω-hydroxyemodin, 3’-hydroxyversiconol) have been screened in this study, but they were reported to show only a modest inhibition of tau aggregation.

In another study by Cornejo et al. the AQ parietin (**68**), extracted from *Ramalina terebrata*, showed to inhibit tau oligomerization in vitro. Docking studies proposed the putative binding mode of parietin to tau protein, suggesting a negative charge density in the inhibitor structure as key feature for targeting some specific lysine residues of tau fibril-forming motifs ^306^VQIVYK^311^ [163].

Purpurin (**69**, 1,2,4-trihydroxyanthraquinone), obtained from the roots of the madder plant (*Rubia tinctorum*) was able to inhibit ~ 50% of PHF6 fibrillization in vitro at equimolar concentration (PHF6: Purpurin) and disassembled pre-formed PHF6 fibrils. Maximum inhibition occurred at a molar ratio of 1:5, which accounted for~90% inhibition. Viswanathan et al. also demonstrated that purpurin ameliorated the AD-like neurodegenerative symptoms and rescued neurotoxicity of *h*Tau in a transgenic fly model. Ex-vivo assays with SH-SY5Y human neuroblastoma cell line overexpressing *h*Tau showed that purpurin effectively reduced the accumulation of the protein [164]. On the base of this findings, purpurin has been proposed as an attractive lead molecule for AD drug development and other related tauopathies.

Finally, the EtOAc extract of the lichen *Xanthoria ectaneoides* have been tested for its potential tau aggregation inhibition and allowed the identification of two promising AQs. Among the two identified derivatives, the only active compound in reducing tau aggregation and promoting fibrils disassembly was the 2-hydroxy-3-[(8-hydroxy-3-methoxy-6-methylanthraquinonyl)oxy]-propanoic acid (**70**) which acts *via* interaction with two specific cysteine residues Cys291-Cys322 that are mainly involved in the polymerization process of tau [165,166].

### 4.3. AQs from Natural Sources: BACE1 Inhibitors and Antioxidants

Beta-secretase 1 (BACE1) is the major β-secretase involved in APP cleavage which determines amyloid-β formation in AD. Thus, the inhibition of BACE1 could be considered as a potential target for the discovery of novel molecules for the treatment of AD.

Jung et al. tested the BACE1 inhibitory potential of the AQs extracted from *Cassia obtusifolia* and discovered the promising BACE1 inhibitory activity of alaternin (**49**, IC_50_ = 0.94 ± 0.04 μg/mL) and emodin (**46**, IC_50_ = 4.48 ± 0.09 μg/mL) [158]. Additionally, 2-hydroxyemodin-1-methylether (**55**), aloe-emodin (**56**), questin (**52**), chryso-obtusin (**54**), and some glucoside analogues (chryso-obtusin-2-O-β-D-glucoside, gluco-obtusifolin and chrysophanol triglucoside) displayed BACE1 inhibitory activities with IC_50_ values ranging from 13.5 to 49.7 μg/mL. Molecular docking calculations on alaternin within the active site of BACE1 allowed to highlight some key residues involved in the ligand-protein interaction; alaternin established hydrogen bond (HB) interactions by mean of its two hydroxyl groups at C(1) and C(10) with Ser36, Asn37, and Ile126 residues of the enzyme, whereas the methyl group in 3 position is involved in hydrophobic interactions with a Tyr198 residue. The docking pose of emodin showed a different HB interaction between Asp32 residue of the enzyme and the hydroxyl group at C(8) of emodin, while its methyl group participated in hydrophobic interactions with five enzyme residues: Trp76, Val69, Phe108, Ala39, and Ile118 [158].

The aforementioned *Morinda officinalis* extracts were also tested for their AD-related activity against BACE1 enzyme. Compounds **60**, **63** and **65** of Figure 13 demonstrated a good inhibitory profile against BACE1 in vitro (IC_50_ = 9.29 ± 1.92 μM, IC_50_ = 25.89 ± 2.11 μM and IC_50_ of 19.82 ± 3.05 μM, respectively), proving similar or higher potency than that of the reference compound quercetin (IC_50_ = 22.75 ± 1.20 μM). Regarding BACE1 inhibitory trend, AQs with only one substituent, such as compound **60** resulted to be more active than the other analogues (**58**, **59**, and **61**–**65**) bearing a greater number of substitutions. The OH group was proven as the best substitution for activity, in particular on C-1, 2, or 3 of AQ scaffold; moreover, three-substituted compounds provided greater activity than derivatives bearing two substituents [161], probably in virtue of the capacity of establishing more effective interaction with the enzyme.

### 4.4. Synthetic AQ Derivatives 

Alongside the discovery of the several potential applications of natural AQ derivatives in treating AD, synthetic analogues have been also developed, with the aim of probing the chemical space around the AQ scaffold that is well tolerated for targeting diverse factors implicated in AD pathogenesis, also simultaneously by exploiting the MTDL strategy.

**Figure 15:**
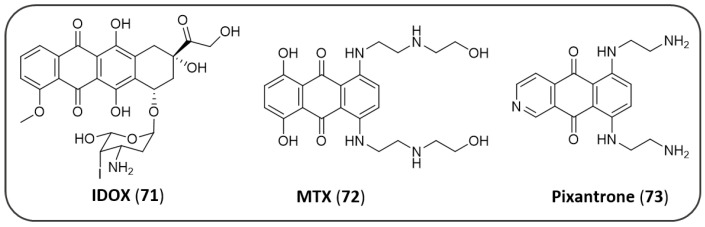
Representative AQ-containing molecules as agents for the treatment of AD.

Some antitumor AQ-based compounds, such as rubicins and xanthrones (Figure 15), have been reported as endowed with activity against some factors implicated in AD. The anthracycline antibiotic 4’-deoxy-4’-iododoxorubicine (**71**, IDOX) demonstrated to reduce the AD amyloid accumulation by binding amyloid deposits and promoting their degradation and resorption [167]. The synthetic AQs mitoxantrone (**72**, MTX) and pixantrone (**73**) also demonstrated to inhibit fibrillogenesis of Aβ_42_ in ThT fluorescence assay (pixantrone: IC_50_ = 26 ± 4 μM) [168].

**Figure 16:**
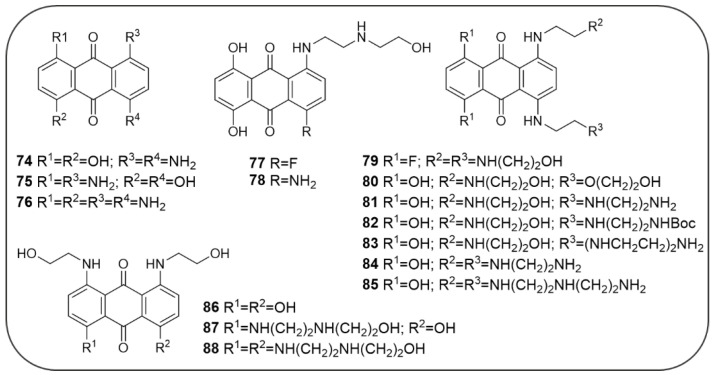
Yang et al. MTX analogues.

Mitoxantrone have also been reported to being able to specifically bind a stem-loop structure in the pre-splicing 4R tau, stabilizing the structure, thus reducing tau aggregation and NFT formation. Yang, L. et al. synthesized a series of MTX (**74**–**88**) analogues in order to clarify the nature of the interaction of MTX with the tau stem-loop domain (Figure 16) [169].

Thus, the binding affinity to pre-mRNA stem-loop tau and stabilizing activity of the analogues have been determined: the insertion of two side chains, as showed by analogues **79–85** and **86–88**, yielded an increased or comparable activity to that of MTX (MTX, binding affinity: EC_50_ = 0.89 µM; stabilizing activity: IC_50_ = 0.46 µM), while for the analogues (**74–76**) devoid of a side chain or decorated with only one side chain (**77**, **78**), the activity slightly decreased or even extinguished. Compound **85**, bearing two side chains functionalized with a polyaminoethyl motif, resulted in a 7-fold increase in binding potency and a 3-fold increase in stem-loop stabilization compared to MTX (**85**, binding affinity: EC_50_ = 0.13 µM; stabilizing activity: IC_50_ = 0.13 µM) [169].

**Figure 17:**
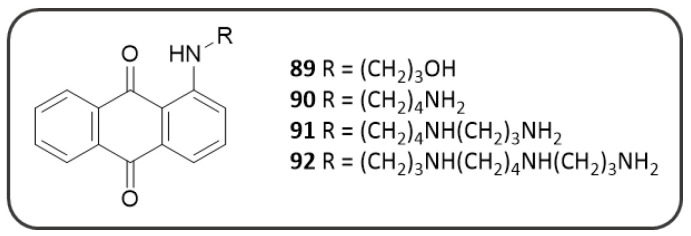
Hong, C. et al. aromatic-polyamine derivatives as ChE inhibitors.

To date, no data are available regarding a potential activity of MTX against the cholinesterase enzymes, however the anti-ChE activity of a series of related AQ-polyamine conjugates (AQ-PCs) have been described by Hong C. et al. (**89–92**, Figure 17), suggesting also for MTX an additional mechanism of action that will deserve further investigations [170].

AQ-PCs (**89**–**92**) demonstrated a good AChE inhibitory activity, while being substantially inactive against BChE. Compounds **90** and **91** resulted to be the best AChE inhibitors with IC_50_ values of 1.50 µM and 2.63 µM, respectively. Docking calculations suggested the possible binding mode of these compounds, encompassing both the PAS and CAS sites: a protonated NH_2_ of the polyamine chain takes contacts to PAS by means of a п-cation interaction with Trp279, while the aromatic tricyclic scaffold establishes п-п stacking interactions in the CAS [170].

**Figure 18:**
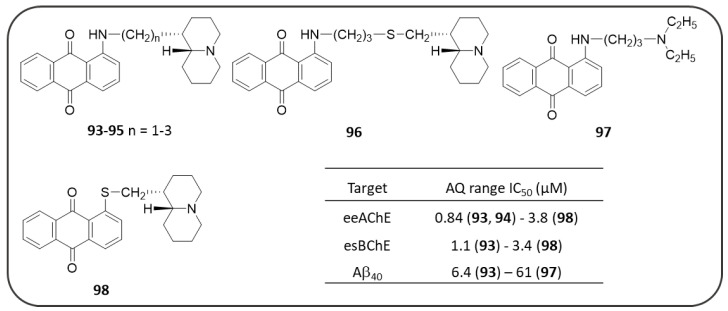
AQ derivatives studied by Sparatore et al. as ChEs and Aβ aggregation inhibitors.

As mentioned in the NQ section (Figure 8), Sparatore F. et al. also described in their work a series of AQs (**92**–**97,** Figure 18) bearing different basic side chains (dialkylaminoalkyl or quinolizidinylalkyl moieties) connected to the AQ scaffold through different linkers of variable length (from 1 up to 5 atoms) [125]. These AQs displayed a dual inhibitory profile towards both *ee*AChE and *es*BChE enzymes, with low micromolar or sub-micromolar IC_50_ values (Figure 18), while provided a slightly lower inhibition of Aβ_40_ aggregation (IC_50_ in the range 6.4–61µM), thus fulfilling the fundamental requisite for a multitarget mechanism of action [125].

**Figure 19:**
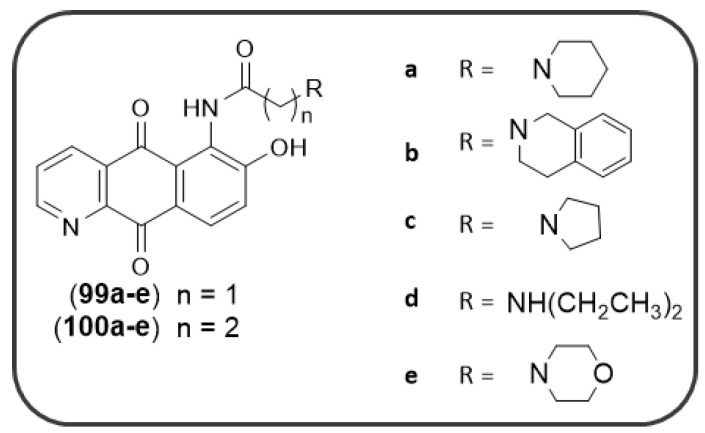
Wang, J. et al. synthetic azaanthraquinones as MTDLs.

Wang, J. et al. recently described the potential activity of a series of synthetic azaanthraquinone compounds (series **99** and **100**, Figure 19) for the treatment of AD, where different substitutions in position 6 of the scaffold were explored [171]. These compounds were found to interfere with different key target points of AD neurodegeneration. They showed the ability to block Aβ_42_ aggregation and secretion, and particularly six compounds, bearing the piperidine or tetrahydroisoquinoline or pyrrolidine rings, shared a potency trend comparable to the reference compound curcumin (% inhibition at 100 µM = 48.1% ± 2.7 µM). Moreover, they displayed anti-inflammatory properties by suppressing NO and iNOS production, and by modulating the synthesis of cytokines. Meanwhile, these azaanthraquinones proved to be AChE-preferring inhibitors with respect to BChE with micromolar IC_50s_ (the most active compounds were the piperidine derivatives **99a** and **100a**: AChE IC_50_ = 1.08 and 1.12 µM, respectively), and to permeate the blood-brain barrier in vitro. Other promising properties included a low degree of toxicity and a neuroprotective efficacy against H_2_O_2_-induced neurotoxicity towards SH-SY5Y neuroblastoma cells [171].

**Figure 20:**
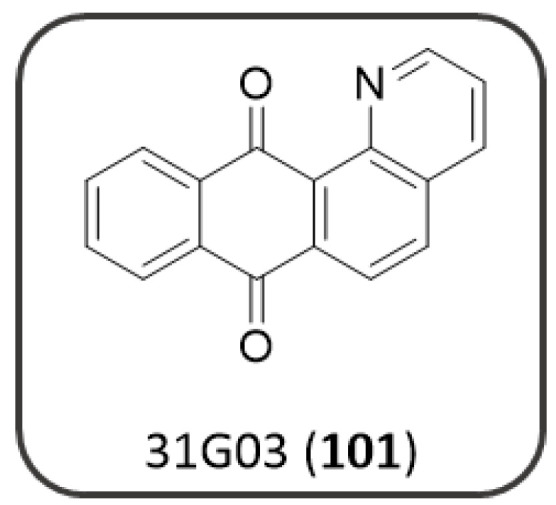
Chemical structure of AQ 31G03.

Finally, high throughput screening (HTS) technology was applied on a heterogeneous compound library in order to discover novel tau aggregation inhibitors and allowed the identification of a single AQ-based compound (**101**, 31G03 of Figure 20) which demonstrated an IC_50_ of 0.63 µM in vitro. This compound may represent an interesting hit worthy of further structural optimization towards the development of improved agents for the treatment of AD [172].

### 4.5. AQ-Based Hybrids

AD progression is characterized by the concurrence of different pathological events occurring in parallel, such as Aβ aggregation, tau hyperphosphorylation and oligomerization, synaptic dysfunctions, and inflammation. Thus, the synthetic efforts in drug design are recently focused on the possibility of including in the same molecule structural features that could hit different key point targets of AD at the same time.

**Figure 21:**
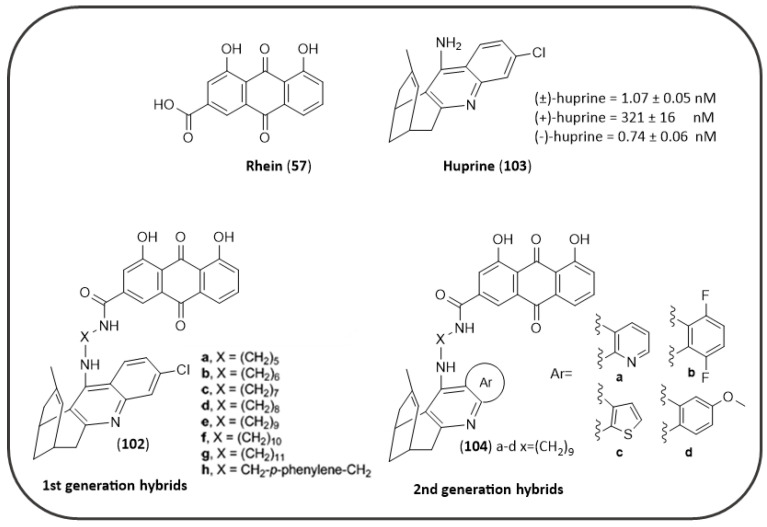
Representative rhein-huprin hybrids.

The natural occurring AQs, such as the aforementioned emodin (**46**) and rhein (**57**), showed an interesting dual inhibitory activity against *h*AChE and tau, thus their scaffolds have been selected as prototypes in order to design novel more efficient multitarget drugs. Since, rhein (IC_50_(AChE) = 18.1 μM [159]) showed only a marginal inhibitory activity towards *h*AChE but possessed the analogous chemical features shared by the previously identified tau oligomerization AQ blockers (see MTX analogues of Figure 16), Viayna et al. developed a series of rhein hybrids (**102a–h**) [173] linked by different spacers to huprine (**103**, Figure 21), a very potent AChE inhibitor (K_I_(AChE) = 24 pM) that is able to establish a specific interaction with the enzyme catalytic anionic site (CAS) [174].

The hybrids have been tested as racemic mixtures and demonstrated potent *h*AChE inhibition, with IC_50_ values falling down in the low nanomolar range. The hybrids presented linkers of different length and nature, thus allowing a careful analysis of the SAR. The inhibitory potency decreased gradually from the compound with the pentamethylene linker (**102a**) to the longest undecamethylene analogue (**102g**). The introduction of a planar and more rigid aromatic ring within the linker produced a negative effect on the inhibitory potency towards the enzyme. The best AChE inhibitor, **102a**, showed an IC_50_ of 1.07 nM, comparing favorably to that of the reference compound huprine. Docking studies revealed that the (−)-hybrids generally bind to the enzyme better than (+)-derivatives taking simultaneous contacts with both CAS and PAS sites: the huprine moiety takes place into the CAS site, while the AQ ring establishes weaker contacts with the PAS. These derivatives were also screened for their activity against Aβ aggregation and BACE1 enzyme, demonstrating also in these cases a potency in the nanomolar range [173].

Starting from the best hybrid of the series (**102a**), a second generation of hybrids have been developed (**104a**–**d**, Figure 21) [175], by varying the structure of the chlorobenzene ring of the huprine moiety with different aromatic or heteroaromatic rings, in order to better understand the structural determinants that mediate the interaction of the huprine moiety of these hybrids to the catalytic site of BACE1. However, this kind of substitutions resulted as less efficient in the inhibition of AChE or BACE1 enzymes, even though they retained or displayed increased potencies as Aβ_42_ and tau antiaggregating agents. Additionally, the [1,8]-naphthyridine (**104a**) or thieno [3,2-e]pyridine (**104c**) hybrids exhibited a potent antioxidant activity, superior to that of the known antioxidants trolox and gallic acid, thus resulting overall the most promising MTDLs [175].

**Figure 22:**
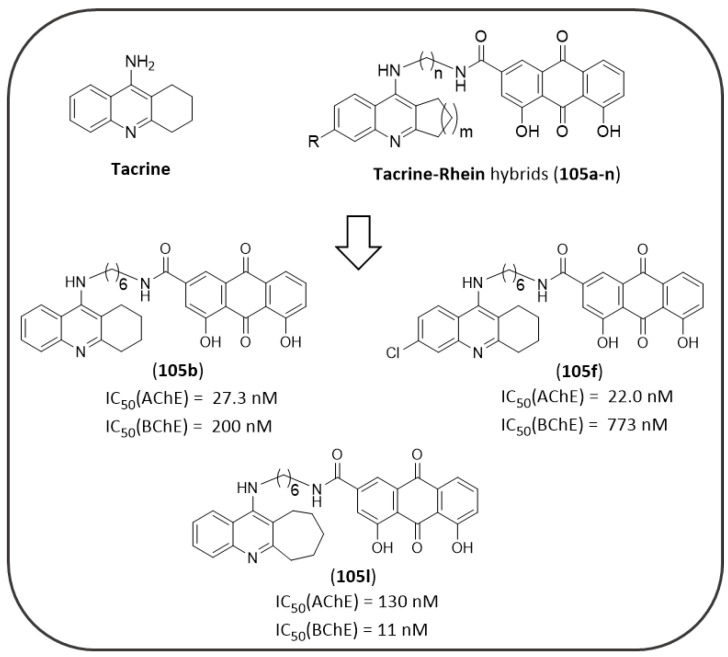
Representative tacrine-rhein hybrids.

The same approach also represented the foundation for the development of a different series of rhein hybrids by Li S.Y. et al. (**105a**–**n**, Figure 22), who combined the AQ scaffold with the anti-cholinesterase drug tacrine [176]. Tacrine has been the first drug approved for the treatment of AD as anti-cholinesterase agent, capable of inhibiting both AChE and BChE. The therapeutic application of this drug has been hampered by the emergence of severe adverse effects, such as hepatotoxicity. Thus, the design of these rhein-tacrine hybrids have been guided by leveraging the potent ChEs inhibition of tacrine, which selectively binds the CAS site of the enzymes, in combination with the metal-chelating, hepatoprotective effects as well as the ChEs inhibitory activity of rhein scaffold, that thanks to its aromatic character could interact with the PAS. Structural modification on tacrine nucleus were focused on the 6-position substitution and the size of its carbocyclic ring, also exploring the length of the polymethylene linker between the two scaffolds [176].

The two most active compounds in inhibiting the *h*AChE were **105b** (IC_50_(AChE) = 27.3 nM; IC_50_(BChE) = 200 nM) and **105f** (IC_50_(AChE) = 22.0 nM; IC_50_(BChE) = 773 nM). The SAR clearly showed that the best size for the carbocyclic ring of tacrine was represented by the six terms ring, as well as the best suited spacer anchoring the two scaffolds seemed to be 6 carbons. In comparison with tacrine, the two hybrids showed an enhanced inhibition of AChE and a reduced ability to block BChE activity (tacrine: IC_50_(AChE) = 135 nM; IC_50_(BChE) = 45 nM). A similar inhibitory profile to that of tacrine was observed for cycloheptyl derivative **105l** (R = H, m = 3, *n* = 6; IC_50_(AChE) = 130 nM; IC_50_(BChE) = 11 nM), which was featured by the highest selectivity profile for BChE. The molecular modeling studies revealed the binding mode of the hybrid **105b** inside AChE active site (PDB:2CKM), where the tacrine moiety was bound to the CAS interacting with Phe330 and Trp84, while the rhein moiety established π–π stacking interactions with Trp279 and Tyr70 of PAS [176]. The occupancy of the AChE’s PAS by the hybrids, as predicted by docking simulations, motivated the authors to assess their capability to inhibit AChE-induced amyloid fibrillation, which derives from the formation of a stable AChE–Aβ complex involving some hydrophobic residues of PAS [177]. Accordingly, all the hybrids presented a strong inhibitory activity on AChE-induced Aβ aggregation, with **105b** providing the greatest potency value (70.2% at 100 μM). Collectively, the multifunctional effects of these hybrids enabled them as potential drug candidates for the treatment of AD, deserving further research.

## 5. Conclusions

The cause of Alzheimer’s disease is still unknown, and the discovery of factors related to the key pathophysiological hallmarks of AD has not been able to uncover the source of the neurodegenerative processes observed in patients. The currently available treatments do not allow long-lasting cytoprotection of nervous cells and appear to be only symptomatic with limited efficacy and perturbing adverse effects [178,179]. To date most pharmaceutical approaches aimed at modifying a single pathological pathway (e.g., cholinergic dysfunction, Aβ and/or tau aberrant processing) have provided an unsatisfactory response [180]. Therefore, from the medicinal chemistry point of view, there is a need of identifying novel chemotypes with a view to formulating medicaments able to ensure a more positive disease outcome over current therapy. Phytochemicals from medicinal plants and other sources are getting attention, as they may provide a valuable alternative to synthetic molecules. During the last years, the ongoing search of 1,4-quinone-based structures, namely NQ and AQ derivatives, have favored a deepening insight of their potential also for treatment of AD. These aromatic bi- and tricyclic systems have demonstrated their relevant contribution to the activity as main structures or important substructures. Moreover, they showed the ability to tackle single or, even more, multiple factors, including ChEs, Aβ and tau proteins, as the most frequently affected targets, thus confirming a multitarget profile, that enables them as valuable candidates for AD therapy. Bulk of evidence supports the multitarget directed ligand (MTDL) approach as an efficient tool to get around the problem of drug-drug interaction and to reduce the risk of toxicity that occur during polypharmacotherapy [181]. The greatest results have been obtained by following a conjugation approach, that has allowed the yield of hybrid molecules composed of a NQ- or AQ-based derivative in combination with another relevant structure, merged or tethered through a linker of variable length and nature, in order to integrate their properties for a more effective treatment of AD. The new SAR insights gathered from this review provide crucial information for the development of more promising options for AD therapy towards the setting up of a drug candidate based on NQ and AQ scaffolds. However, most of the research in this context has limited the study of biological evaluation to an early stage, mainly at enzyme and cellular level, and a few studies have explored the drug-like properties of these molecules, thus further efforts are deserved, before they could be translated into therapeutics for an effective AD management. 

## Data Availability

Not applicable.
